# Synthetic Bone Substitute Engineered with Amniotic Epithelial Cells Enhances Bone Regeneration after Maxillary Sinus Augmentation

**DOI:** 10.1371/journal.pone.0063256

**Published:** 2013-05-17

**Authors:** Barbara Barboni, Carlo Mangano, Luca Valbonetti, Giuseppe Marruchella, Paolo Berardinelli, Alessandra Martelli, Aurelio Muttini, Annunziata Mauro, Rossella Bedini, Maura Turriani, Raffaella Pecci, Delia Nardinocchi, Vincenzo Luca Zizzari, Stefano Tetè, Adriano Piattelli, Mauro Mattioli

**Affiliations:** 1 Department of Comparative Biomedical Science, University of Teramo, Teramo, Italy; 2 Department of Technologies and Health, Istituto Superiore di Sanità, Rome, Italy; 3 Department of Surgical and Morphological Science, University of Insubria, Varese, Italy; 4 Department of Medical, Oral and Biotechnological Science, University “G. d'Annunzio”, Chieti, Italy; 5 Stem TeCh Group, Chieti, Italy; The Ohio State University, United States of America

## Abstract

**Background:**

Evidence has been provided that a cell-based therapy combined with the use of bioactive materials may significantly improve bone regeneration prior to dental implant, although the identification of an ideal source of progenitor/stem cells remains to be determined.

**Aim:**

In the present research, the bone regenerative property of an emerging source of progenitor cells, the amniotic epithelial cells (AEC), loaded on a calcium-phosphate synthetic bone substitute, made by direct rapid prototyping (rPT) technique, was evaluated in an animal study.

**Material And Methods:**

Two blocks of synthetic bone substitute (∼0.14 cm^3^), alone or engineered with 1×10^6^ ovine AEC (oAEC), were grafted bilaterally into maxillary sinuses of six adult sheep, an animal model chosen for its high translational value in dentistry. The sheep were then randomly divided into two groups and sacrificed at 45 and 90 days post implantation (p.i.). Tissue regeneration was evaluated in the sinus explants by micro-computer tomography (micro-CT), morphological, morphometric and biochemical analyses.

**Results And Conclusions:**

The obtained data suggest that scaffold integration and bone deposition are positively influenced by allotransplantated oAEC. Sinus explants derived from sheep grafted with oAEC engineered scaffolds displayed a reduced fibrotic reaction, a limited inflammatory response and an accelerated process of angiogenesis. In addition, the presence of oAEC significantly stimulated osteogenesis either by enhancing bone deposition or making more extent the foci of bone nucleation. Besides the modulatory role played by oAEC in the crucial events successfully guiding tissue regeneration (angiogenesis, vascular endothelial growth factor expression and inflammation), data provided herein show that oAEC were also able to directly participate in the process of bone deposition, as suggested by the presence of oAEC entrapped within the newly deposited osteoid matrix and by their ability to switch-on the expression of a specific bone-related protein (osteocalcin, OCN) when transplanted into host tissues.

## Introduction

Bone regeneration in maxillary sinus is an essential condition for dental implants in atrophic posterior maxilla. Different strategies leading to the replacement of missing bone have been conventionally used for over 30 years [1], [2]. Limited availability of autografts, and the risk of disease transmission by allo/xenografts, have increased the demand of synthetic bone substitutes, which have to reproduce the physical/chemical properties of native bone tissues in order to maximize osteointegration, osteoconduction and osteoinduction [2]. Calcium phosphate ceramics, such as hydroxyapatite (HA) and tricalcium-phosphate (TCP), are considered both suitable materials for bone reconstruction since they conjugate a high biocompatibility with an efficient osteoconductivity [3]. The porous architecture and the degree of interconnectivity are additional critical factors to determine the clinical success of biomaterials [4], [5]. In fact, the chemical composition and architecture of biomaterials are both crucial to drive and stimulate bone healing and deposition. In order to mimic the structure of native bone and to ensure cell viability and function, the ideal scaffold should exhibit porosity at different length scales: nano-porosity, to allow molecule transport essential for any nutrition, waste removal and signaling; micro-porosity, to ensure cell migration and capillary formation; millimeter-wide porosity to incorporate nerves and blood vessels [6], [7]. Scaffold porosity improves mechanical interlocking between the implanted biomaterial and the surrounding host bone [8], [9], and positively influences the scaffold degradation rate. During the last few years, innovative technologies, such as three-dimensional (3D) printing and dispense-plotting, allowed to create scaffolds with a controlled 3D architecture [9]–[13], thus enhancing their biocompatibility [14]–[17]. However, the latest generation of synthetic bone substitutes still requires a long time to regenerate a large amount of bone tissue thus limiting their surgical use in validated therapeutic protocols such as sinus augmentation [18], [19]. Therefore, cell-based therapies are an emerging strategy to improve bone tissue healing and regeneration [20]–[23]. In this context, increasing attention has been recently addressed to placental components and, in particular, to amnion as a possible reserve of stem/progenitor cells [24]–[29]. Actually, the therapeutic use of amniotic membrane has been studied for decades. Davis first reported in 1910 the use of fetal membranes as surgical materials in skin transplantation performed on 550 patients [30]. Amniotic membranes showed anti-inflammatory [31]–[33], antimicrobial [34], antifibroblastic [35] and low immunogenicity properties [36], [37]. Several surgical applications for amniotic membranes have been reported, including their use as a biological dressing for the treatment of skin wounds, burn injuries and chronic leg ulcers, as well as in the treatment of tissue adhesion in surgical procedures and ocular burns [26].

More recently, amniotic membranes have been investigated as a possible source of stem/progenitor cells for therapeutic applications. Cells from mesenchymal and epithelial amniotic layers, amniotic mesenchymal stromal and amniotic epithelial cells (AEC), respectively, can be obtained without any ethical concerns, in large amounts and with validated protocols [38], [39]. Amnion derived cells have been shown to maintain a remarkable plasticity and to possess a high self-renewing capacity [40]–[45]. To date, AEC have been more extensively investigated and their potential to differentiate into cell types of all three germ layers has been demonstrated [24], [25], . The mesodermal differentiation attitude of AEC has attracted increasing attention [25], [44], [48], [49], in particular, by considering the usefulness of amniotic derived cells in supporting bone lineage differentiation [25], [26], [28], [29]. Moreover, *in vitro* and *in vivo* studies demonstrated how also isolated AEC maintain no-immunogenic and even immunosuppressive properties [50]–[52] Even more important, AEC are not carcinogenic after transplantation [53] thus allowing a safe use in allogeneic preclinical settings into immunocompetent animals [44], [47], [54]–[58].

Based on previous findings, the present research has been designed to evaluate whether AEC may represent an alternative source of progenitor/stem cells for cell-based therapeutic protocols in dentistry. To this aim, their regenerative properties were studied in an animal model. In detail, ovine AEC (oAEC) were engineered on a custom-made 3D hydroxyapatite (HA) and beta TCP (β-TCP) scaffold and grafted into the maxillary sinus of six adult sheep, before evaluating their influence on bone regeneration at 45 and 90 days post implantation (p.i.).

## Materials and Methods

### Chemical Agents

All chemical reagents and media were purchased from Sigma Chemical Co (St Louis, MO, USA), unless otherwise specified.

### Ovine AEC Isolation And Characterization

#### Ovine AEC Isolation

As previously described, oAEC were obtained by enzymatic digestion from five sheep slaughtered at approximately 3 months of pregnancy [44]. Freshly isolated oAEC were seeded in flasks containing minimum essential medium eagle alpha (αMEM), supplemented with 20% fetal calfserum (FCS), 1% ultraglutamine, 1% penicillin/streptomycin plus 10 ng/ml epidermal growth factor (EGF) at a concentration of 3×10^3^ cells/cm^2^. At 70–80% confluence, cells were dissociated by 0.05% trypsin-ethylenediaminetetraacetic acid (EDTA) and plated at the same concentration (3×10^3^ cell/cm^2^) for 3 consecutive passages. At the end of expansion, oAEC were stored (2.5×10^6^ cells/vial) in liquid nitrogen before using For each pool of stored oAEC belonging to each fetus, some vials were randomly thawed to test the maintenance of the following biological properties before using the remaining batch of cells in animal model. More in detail, aliquots of frozen oAEC were assessed for:

- cytokeratin 8 (epithelial marker) and alpha smooth muscle actin (α-SMA: mesodermal marker), expression by immunocytochemistry [46];
- hemopoietic, adhesion, pluripotent and major histocompatibility complex (MHC) markers expression by cytofluorimetry;
- the ability to differentiate into three different mesodermal cell lineages (osteoblast, tenocytes and adipose cells) by molecular biology and immunocytochemistry.

#### Immunocytochemical Detection Of Cytokeratin 8 And αSMA

Immunocytochemical analyses were carried out on freshly isolated, expanded and thawed oAEC as previously described [46]. Briefly, cells were fixed in 4% paraformaldehyde in phosphate buffered saline solution (PBS) and permeabilized with 0.1% Triton X-100/PBS. After incubation in PBS/1% bovine serum albumine (BSA) for 1 hour, oAEC were incubated with primary and secondary antibodies (Abs, see Table 1 for details). Negative controls were obtained by omitting the primary Ab. Cell nuclei were counterstained with 4′-6-diamidino-2-phenylindole dihydrochloride (DAPI).

**Table 1:** Details of antibodies used in immunofluorescent analysis.

Primary Abs (Company details)		Secondary Abs (Company details)	
	µg/ml		µg/ml
**Cytokeratin 8**	5	**Anti-Mouse Cy3**	2
(Abcam, Cambridge, UK)		(Sigma-Aldrich, Missouri, USA)	
**vWF**	0.02	**Anti-Rabbit FITC**	5
(Dako, Glostrup, Denmark)		(Sigma-Aldrich, Missouri, USA)	
**α-SMA**	4	**Anti-Mouse Alexa Fluor488**	5
(Abcam, Cambridge, UK)		(Sigma-Aldrich, Missouri,USA)	
**Osteocalcin**	20	**Anti Mouse Alexa Fluor488**	5
(Abcam, Cambridge, UK)		(Invitrogen, Oregon, USA)	
**VEGF**	100	**Anti-Rabbit Alexa Fluor488**	5
(Calbiochem, USA)		(Invitrogen, Oregon, USA)	

#### Flow Cytometer Analysis

oAEC were screened immediately after isolation, before and after thawing by flow cytometry, according to Barboni *et al.* [46] and using the Abs reported in Table 2. Abs were used for FACSCalibur flow cytometer (BD Bioscience) analyses and results were elaborated on CellQuest™ software (see Table 2 for details). Flow cytometry measurements were carried out by using rainbow calibration particles (6 peaks) and CaliBRITE beads (both from BD Biosciences) as quality control. Debris was excluded from the analysis by gating on morphological parameters (lymphocyte gate); 20.000 non-debris events in the morphological gate were recorded for each sample. All Abs were titrated under assay conditions and optimal photomultiplier (PMT) gains were established for each channel [59]. Data were analysed using FlowJo™ software (TreeStar, Ashland, OR). Mean fluorescence intensity ratio (MFI) was calculated dividing the MFI of positive events by the MFI of negative events [60].

**Table 2:** Details of primary and secondary antibodies used in flow cytometry analysis.

Antigen	Conj. fluorescent probe	Company details	
***Hemopoietic markers***			
CD14	FITC	LifeSpan Biosci.	Seattle, WA, USA
CD31	FITC	AbD Serotec	Oxford, UK
CD45	FITC	AbD Serotec	Oxford, UK
***Adhesion molecules***			
CD29		VMRD	Pullman, WA, USA
CD49f		Beckman Coulter	Fullerton, CA, USA;
CD166	FITC	Ancell	MN, USA
***MHC antigens***			
Class I		Novus Biologicals	Cambridge UK
Class II HLA-DR		Abcam	Cambridge, UK
***Stemness markers***			
CD117		Abcam	Cambridge, UK
SOX2		Abcam	Cambridge, UK
OCT4	PE	Becton Dickinson	BD, San Jose, CA
TERT		Calbiochem	Gibbstown, NJ
NANOG		Chemicon Int.	Billerica, MA

#### Mesodermal Lineage *In Vitro* Differentiation

oAEC were preliminary differentiated *in vitro* into three different mesodermal cell lineages. Osteogenic differentiation was obtained according to Mattioli *et al.* [44]. Briefly, oAEC (2×10^4^ cells) were incubated in osteo-inductive medium (αMEM plus 50 µM ascorbic acid, 10 mM β-glycerol phosphate, 0.2 µM dexamethasone and 10% FCS) for 21 days and osteogenic differentiation was evaluated by Alizarin Red staining [44] and RT-PCR (see Table 3 for details). Tenogenic *in vitro* differentiation was performed co-culturing oAEC with fetal tendon explants isolated from calcaneal tendons, as previously described [46]. Tenogenic differentiation was monitored by analyzing oAEC ability to generate three dimensional tendon-like structures enriched in collagen type I (COL1), as well as to express specific tendon-related-genes (see Table 3 for details). Finally, adipogenic differentiation was investigated by culturing oAEC in αMEM supplemented with 10% FCS, 0.5 mM isobutyl-methylxanthine (IBMX), 200 µM indomethacin, 0.1 µM dexamethasone, 10 ug/ml insulin and 1 µM rosiglitazone/BRL49653 for 4 weeks, according to a validated method [61]. For adipogenesis evaluation, cells were stained with oil-red O and, in parallel, tested with RT-PCR to evaluate the mRNA content for two adipose-related genes (see Table 3 for details).

**Table 3:** Primer sequences used in real time and RT-PCR analyses.

Gene	Accession No.	Primer sequences	size (bp)	Cycles
***COL1*** b,t	AF129287.1	F: CGTGATCTGCGACGAACTTAA	212	40
	Ovine	R: GTCCAGGAAGTCCAGGTTGT		
***SCXB*** t	XM_866422.2	F: AACAGCGTGAACACGGCTTTC	*299*	*45*
	Bos Taurus	R: TTTCTCTGGTTGCTGAGGCAG		
***OCN*** b,t	DQ418490.1	F: AGACACCATGAGAACCCCCAT	234	40
	Ovine	R: TTGAGCTCACACACCTCCCT		
***LPL*** a	NM_001009394.1	F: GTCACGGGCCCAGCAGCATT	313	40
	Ovine	R: GCCAGGTGACCCCCTGGTGA		
***PPAR*** a	AY137204.1	F: CGCATGCCACAGGCCGAGAA	265	40
	Ovine	R: CCTGCAGGGGGCTGATGTGC		
***TNMD*** t	NM_001099948.1	F: GTCACGGGCCCAGCAGCATT	352	40
	Bos Taurus	R: GCCAGGTGACCCCCTGGTGA		
***GAPDH*** *	AF030943.1	F: CCTGCACCACCAACTGCTTG	224	40
	Ovine	R: TTGAGCTCAGGGATGACCTTG		
***VEGF*** *	AF250375.1	F: GTGCCCACTGAGGAGTTCAA	208	40
	Ovine	R: GTCTGGTTCCCGAAACCCTG		

b,t,a superscripts indicate specific tissue-related genes expressed in bone, tendon and adipose tissues, respectively.

* primers used in real time PCR to quantify the VEGF expression. The other primers were used instead under RT-PCR.

#### Total RNA Isolation And RT-PCR

Total RNA was extracted from oAEC, native tissues (bone, tendon and adipose tissue) and from cryosections of explanted sinuses by using TRI Reagent, followed by reverse transcription (RT) reaction, as previously described [46]. RT-PCR was performed in order to define the expression profile of specific genes (Table 3). PCR products were separated on 2% agarose gel stained with ethidium bromide, visualized on a Gel Doc 2000 (Bio-Rad Laboratories) and analyzed with Quantity One 1-D Analysis software (Bio-Rad Laboratories). RT-PCR was normalized by the transcriptional levels of glyceraldehyde 3-phosphate dehydrogenase (GAPDH). Each PCR reaction was carried out in triplicate.

#### Ovine AEC Labeling

Thawed oAEC displaying a stable proliferation index, phenotype and the ability to undergo mesodermal lineage differentiation were then used for the animal model experiments described below. Before performing sinus augmentation, oAEC were marked with a fluorescence lipophilic dye, the PKH26, as previously described [44], [47], [62].

### Biomaterial Characterization

The ceramic scaffolds used in this study were fabricated by the direct rPT dispense-plotting. A virtual scaffold model was designed with a cylindrical outer geometry by using 3D computer aided design software. The size of the model was adapted to the shrinkage of the ceramic material in the subsequent sintering process. The inner geometry, i.e. the pathway of the material rods, was defined by custom-made software which generates the control commands of the rapid prototyping machine. To build up the green bodies, material rods, consisting of a paste-like aqueous ceramic slurry, were extruded out of a cartridge through a nozzle and deposited using an industrial robot (GLT Pforzheim, Germany). In this study, HA/β-TCP powders (Merck, Germany) were blended to get a powder mixture with a HA/β-TCP weight ratio of 30/70. The characteristic rheological behavior of the aqueous ceramic slurry was achieved by thermal treatment of the raw HA powder at 900°C for 1 h and by adding a compatible binder/dispersant system of organic additives of 10.5 wt% relative to the mass of ceramic powder. The rod deposition was controlled in x, y and z direction to assemble 3D scaffolds layer by layer on a building platform. By rotating the direction of the rod deposition by 60° from layer to layer a three-dimensional network with an interconnecting pore structure was generated. The assemblies made of ceramic slurry were dried at room temperature and subsequently sintered at 1250°C for 1 h. Finally the sintered scaffolds were reduced to smaller blocks with a volume of about 0.14 cm^3^ in order to remove the solid rim that resulted from the turning points at the edge of the printed pathways. Images of the sintered dispense-plotted scaffolds taken with a light microscope (MZM1, Askania, Mikroskop Technik Rathenow, Germany) were analysed using an image analysis system (analySIS, Soft Imaging System, Germany) to determine rod diameters and pore sizes. Density of the sintered scaffolds was measured using weight/volume method in which volume was determined geometrically and by using a helium pycnometer (AccuPyc 1330, micromeritics, Germany). Semi-quantitative X-ray diffraction measurements (XRD, 3000P, Seifert, Germany), and scanning electron microscopy (SEM; Quanta 200, FEI, The Netherlands) were used to characterize the surface of material rods and fracture faces of sintered samples.

### Ovine AEC Scaffold Loading

In order to achieve 3D scaffold loading, 1×10^6^ PKH26-labeled oAEC were seeded on single blocks of synthetic bone substitute (∼0.14 cm^3^) and incubated in 35 mm Petri dish over a roller apparatus (Wheaton, Millville, NJ). Each culture was taken under agitation for 3 days at a speed of 6 rpm. In order to verify the efficiency of loading process, the incubation medium was collected and centrifuged to quantify the concentration of unloaded cells. In addition, samples of HA/β-TCP biomaterials were, preliminarily, incubated with 1×10^6^ oAEC to evaluate the incidence and the distribution of vital cells on the biomaterial surface. To this aim, the vital cytoplasmic fluorescent dye Calcein-AM was used [44]. The scaffolds used in control group (CTR) were incubated without cells for 3 days under analogous cultural conditions.

### Animal Model

#### Animals

The present study was approved by the Ethics Committee of the Universities of Teramo and Chieti-Pescara (prot. 05/2012/CEISA/PROG/32). Six adult sheep, two years old and 40–50 Kg of weight, were bred according to the European community guidelines (E.D. 2010/63/UE) before performing bilateral sinus augmentation. Animals were quarantined for 2 weeks to check the general healthy status. Surgical procedures were then carried out in an authorized veterinary hospital. The animals were randomly divided into two groups and euthanized to explant grafted sinuses at 45 and 90 days p.i..

#### Sinus Augmentation Surgical Procedure

Sheep were operated under general anesthesia to carry out a bilateral sinus augmentation as previously described in Berardinelli *et al* [63]. The lateral wall of the sinus was approached through an oval ostectomy (1 cm up and 1 cm caudal to tuber facial tuberosity) carried out using a piezoelectric unit (Biosafin Easy Surgery, Italy). The *Schneiderian* membrane was elevated and the cavity between the mucosa and the inferior osseous septum of the sinuses was augmented with two blocks of biomaterial alone (CTR) or previously loaded with PHK26 labeled oAEC. The animals were treated i.v. with 20 mg/kg of ampicillin (Vetamplius®, Fatro) every 12 h for 3 days. Surgical wounds were inspected daily. Groups of three animals were euthanized at 45 and 90 days p.i. by applying an overdose of thiopental (Pentothal Sodium, Intervet) and embutramide (Tanax®, Intervet).

### Morphological Analysis Of Sinus Explants

Sinus explants were fixed overnight in 4% paraformaldehyde solution (PBS, pH 7.4) and then analyzed by micro-CT technique in order to evaluate the biomaterial integration into the host tissue. The fixed explants were then processed for histological, immunohistological and biochemical analyses.

#### Micro-CT analysis

The bi-dimensional (2D) micro-CT analysis was performed using the SkyScan1072 (SkyScan, Belgium) operating at 98 µA currents and 100 kV voltages. The samples were analyzed at 20 mm×20 mm image size reaching a sample thickness of 19.5 µm. Since the large-size explanted samples were acquired at low resolution, in order to compare micro-CT images with the histological sections, no quantitative measures have been obtained.

The data sets were acquired over a rotation range of 180° (with 0.45° rotation step) and reconstructed with a software (NRecon v1.6.3; SkyScan, Belgium) based on the cone beam algorithm. 3D reconstruction was carried out by using ANT 3D Creator (v2.4; SkyScan, Belgium).

#### Histological Analysis

The explants were decalcified for at least 30 days in 14% EDTA [64]–[67] and placed overnight in 30% sucrose-PBS before freezing. Each explant was completely sectioned at 10 µm of thickness. For morphological and morphometric analysis, serial sections were collected every ∼400 µm of distance. Two sections were stained with hematoxylin–eosin (HE) to evaluate the architecture of newly formed tissues and the presence of inflammation. Histological grading of inflammation was carried out by two independent investigators blind to the experimental conditions, using a semi-quantitative scoring system adapted from Cargnoni *et al.* [68]. For each group, at least 6 sections were analyzed. More in detail, 20 fields at ×200 final magnification were assessed for each tissue section under study, and the severity of inflammation was scored from 0 to 3 to reflect absent, mild, moderate or extensive inflammatory cell infiltration. Scores were summed and reported as mean value. For polarized light microscopy, HE-stained sections were observed using two orthogonally polarized light waves (Leica, Switzerland).

### Morphometric and Biochemical Quantitative Analyses

Morphometric and biochemical analyses were performed on at least 10 different serial sections/animals (3 sections for HE, 3 for vascular area, VA, and 4 for vascular endothelial growth factor, VEGF, expression analysis) [69]. Morphometric data were calculated on at least 8 different fields/sections, randomly distributed in the central (C) or peripheral (P) portion of the implanted area, were blind evaluated.

A light microscopy (Axioscop 2 plus microscope) provided with a digital camera (Axiovision Cam, Zeiss, Germany) was used for image capture, while software ImageJ® 1.44 (National Institutes of Health, USA) was used to quantify the extension of newly deposited bone, scaffold and soft tissues within the implanted area. In detail, HE analysis were carried out at ×50 magnification and images obtained were processed to digitally convert scaffold, soft and newly deposited bone tissues in different colors as described in Berardinelli *et al.* [63]. The relative tissues/scaffold extension was calculated on the digitally converted images. The quantification of newly deposited bone was performed exclusively by considering the vital bone as the amount of mineralized and vascularized tissues displaying osteocytes within their lacunae [64], [70]–[71].

In addition, serial sections were immunolabeled by using an anti-von Willebrand factor (vWF) Ab (Table 2), in order to detect blood vessel remodeling and to calculate the total vascular area (VA: see morphometric analyses), according to Martelli *et al.* [69]. In detail, primary Ab (Table 2) was applied at room temperature overnight after hotbox oven at 95°C before exposing the cells to secondary Ab (Table 2). Ovine aorta tissue was used as positive control. Negative controls were obtained by omitting the primary Ab. Tissue sections were counterstained with DAPI to visualize cell nuclei. Blood vessel analyses were carried out by using an Axioscop 2 plus microscope (Zeiss, Germany) and a digital camera (Axiovision Cam, Zeiss, Germany). Data were processed with a KS300 computed image analysis system (Zeiss, Germany), as previously described [69], [70]. The total VA was quantified at ×200 magnification and the digitized fluorescent vessel signals (vWF positive area) were obtained using a semi-automated algorithm [70]. The VA was calculated as the extension expressed in µm^2^ of vWF-positive area/field (15000 µm^2^).

Real time PCR was performed to evaluate the VEGF expression on grafted sinuses at 45 and 90 days p.i.. More in detail, laser capture micro dissection (LCM) was performed using a MMI Cellcut apparatus to isolate the implanted area from native tissues. Frozen sections (n = 12/group) were briefly air dried on the glass slides and then kept on dry ice until they were subjected to LCM. Just before the procedure, the sections were exposed to 70% ethanol for 10 seconds and stained with HE. The settings of the laser were: spot diameter 20 µm, pulse duration 50 ms and power 50 mW. The area to micro dissect was identified under a light microscope at ×40 of magnification. The micro dissected area including the implantation site was dropped onto a separate cap before going on to RNA extraction and RT reaction as previously described. Real-time quantitative PCR was performed in the Stratagene MX3000P instrument using SYBR Green I dye detection. For VEGF and GAPDH genes the primers used were reported in Table 3. The following reaction components were prepared to the indicated end-concentrations: 2.5 µl forward (0.25 µM) primer, 2.5 µl reverse (0.25 µM) primer (Table 3), 3.5 µl water and 12.5 µl Brilliant SYBR Green QPCR Master Mix 2X (Stratagene). 3 µl of cDNA were added to 22 µl of the master mix. The real-time protocol employed was: initial denaturation for 10 min at 95°C, 40 cycles at 95°C for 45 sec, 60°C for 45 sec, 72°C for 45 sec. The specificity of amplicons was confirmed both by dissociation curve and by gel electrophoresis. Each sample was run in triplicate and for quantitation of the VEGF gene target the Comparative *C*t Method was applied for all samples normalized to the control housekeeping GAPDH gene using the formula: 2^−ΔΔ*C(t*)^ = 2 −^(Δ*Ct* control gene^−^Δ*Ct* target gene)^. For statistical analyses the mean of three independent experiments was considered.

### Ovine AEC Detection In Sinus Explants

The PKH26-labelled oAEC were identified on cryosections, by using an Axioskop 2 plus (Zeiss, Germany) epifluorescence microscope (excitation: 551 nm, emission: 567 nm). In order to count the endogenous and transplanted cells, the cryosections were processed with the KS300 computed image analysis system (Zeiss, Germany) by performing a guided program (macros for KS300, Zeiss, Germany) created to count the cells inside a standard field of 15000 µm^2^, at ×200 magnification. In detail, for the present study two classes of cells were considered:

- PKH26 positive oAEC are expressed by the number of cells displaying the DAPI counterstained nucleus surrounded by the red fluorescence PKH26 dye;
- endogenous cells were the remaining cells identified exclusively by the DAPI stained nuclei.

Finally, to evaluate the contribution of oAEC to the process of tissue regeneration the co-labeling between OCN, VEGF or vWF and PKH26 were analyzed on cryosections collected at 45 and 90 days p.i.. Moreover, *in vitro* cultured PKH26 labeled cells were fixed in 4% paraformaldehyde/PBS and permeabilized with 0.05% Tween 20/PBS as described in the previous materials and methods paragraph. After incubation in PBS/1% BSA, OCN or VEGF primary and secondary Ab were applied (Table 2). According to the data sheet instructions, ovine calvaria tissue was used as positive control and the omission of primary Ab as negative control.

### Statistical Analysis

The data were checked for normal distribution by D'Agostino and Pearson test and were compared, after arctan (x) transformation, by two way-ANOVA test to compare the effect exerted by different treatments. Finally, the post-hoc Tukey test (GraphPad Prism 5, GraphPad Software, USA) was carried out in order to evaluate the “individual” effect on each examined variable. The data are reported as mean ± Standard Deviation (± S.D).

## Results

### Ovine AEC Isolation And Molecular Characterization

**Figure 1:**
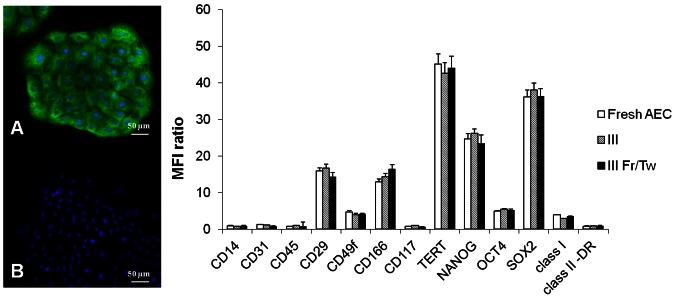
Molecular characterization of cultured and cryopreserved oAEC. Immunocytochemestry investigations show that freshly isolated oAEC displayed cytokeratin-8 positivity (A) while αSMA expression is not detectable (B). oAEC nuclei are counterstained with DAPI (blue fluorescence). Histograms show the average of surface, MHC and pluripotency markers expressed as MFI ratio levels obtained with flow cytometry analysis (3 replicates ± SD). The analyses were performed on oAEC expanded *in vitro* for III passages, before and after freezing and thawing (Fr/Tw).

**Figure 2:**
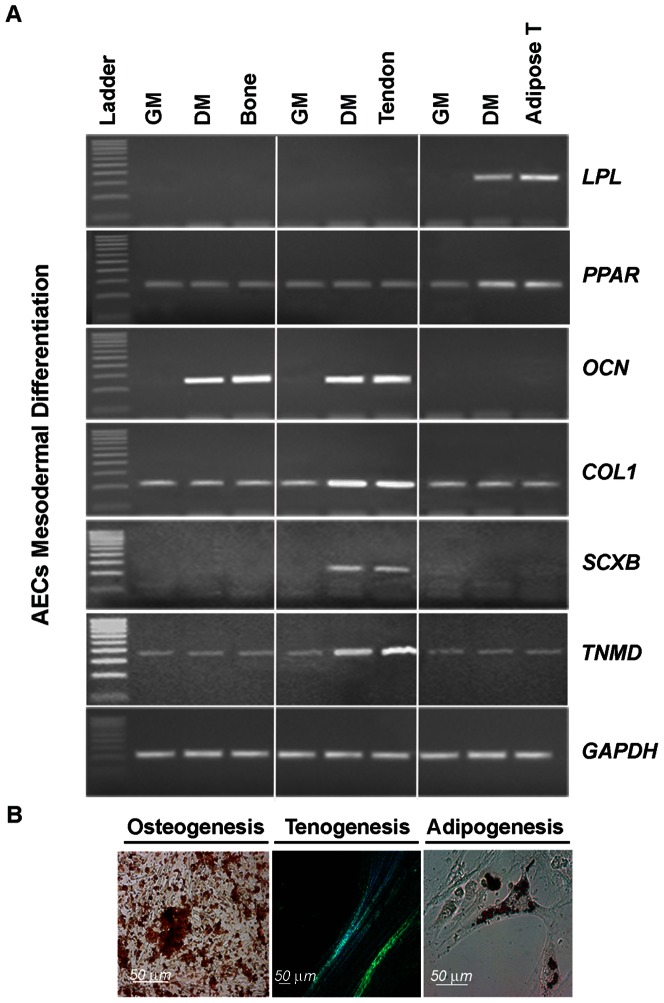
Expression profile of tissue specific-related genes after ***in vitro*** **oAEC mesodermal differentiation.** A) The mRNA content in *LPL, OCN, COL1, SCXB, PPAR, TNMD* and, *GAPDH* was analysed by RT-PCR in oAEC cultured for 28 days in growth medium (GM) or in adipogenic, osteogenic and tenogenic differentiation media (DM). B) Image examples showing the *in vitro* mesodermal differentiation properties of oAEC: left, the mineralization of extracellular matrix evidenced by Alizarin Red staining, middle, COL1 protein (green fluorescence) distribution recorded by immunohistochemistry in cell aggregates obtained during oAEC differentiation toward tenogenic lineage (blue nuclei counterstained with DAPI), right, intracellular lipid droplets evidenced by oil-red O staining.

The oAEC isolated from amniotic membranes were characterized by a uniform cell population characterized by a high cytokeratin 8 expression and by the absence of/low αSMA positivity (Fig. 1AB). The oAEC expanded in *vitro* for 3 passages did not substantially modify their molecular and antigenic profile (Fig. 1) as confirmed by flow cytometry analysis. In detail, oAEC did not display any hemopoietic markers (CD14, CD31 and CD45) and MHC class II antigens (Fig. 1). On the contrary, oAEC expressed several surface adhesion molecules (CD29, CD49f and CD166) and, to a lesser degree, MHC class I antigen. Freshly, expanded and cryopreserved oAEC showed similar levels of pluripotency markers (TERT, SOX2, OCT4 and NANOG; see Fig. 1). Moreover, RT-PCR demonstrated that oAEC had undetectable mRNA level of *SCXB*, *OCN* and *LPL* and, a low expression of *COL1*, *TNMD* and *PPAR* (Fig. 2A). *In vitro* differentiation tests confirmed the mesodermal attitude of oAEC. In detail, osteogeneic differentiation was suggested either by the prompt ability of oAEC to mineralize the extracellular matrix demonstrated by Alizarin Red staining (Fig. 2B) or by the increased levels of *COL1* mRNA and the appearance of *OCN* gene expression (Fig. 2A). Similarly, oil-red O staining (Fig. 2B) and *LPL* and *PPAR* gene expressions (Fig. 2A) demonstrated the ability of oAEC to undergo *versus* adipose tissue lineage. Finally, the oAEC co-cultured with fetal tendon explants underwent tenogenic differentiation developing 3D elongated structures constituted of fusiform aligned cells displaying a high intracellular content of COL1 (Fig. 2B). In parallel, oAEC modified their gene profile displaying an increased expression of tendon related genes such as *COL1*, *TMND* and *SCX* (Fig. 2A).

### Scaffold Characterization and Loading

**Figure 3:**
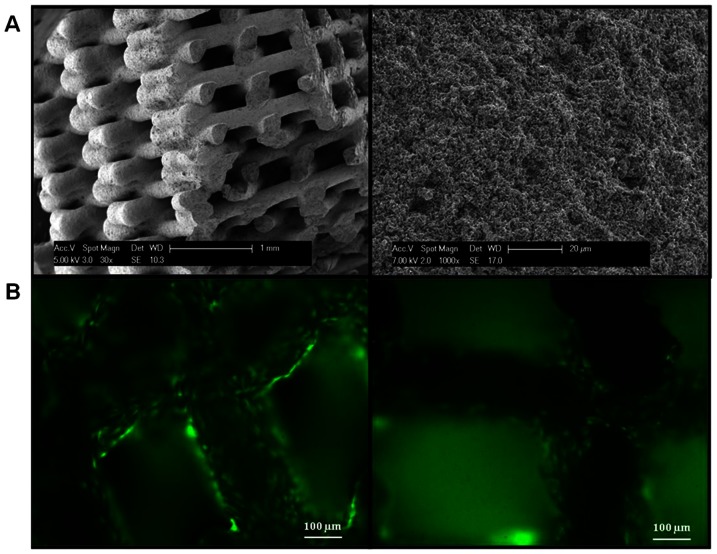
Ovine AEC engineered bone synthetic scaffold. A) Scanning electron microscopy images showing the surface and fracture face of direct rPT HA/β-TCP scaffold in different magnifications. Left image: fracture through ceramic scaffold. Right image: surface (white star) and fracture face (white arrow) of material rod. B) An example of HA/β-TCP scaffold's loaded with 1×10^6^ oAEC under rotating culture for 3 days. The high concentration of cells loaded on biomaterial surface were visualized by the vital fluorescence permeable dye Calcein-AM (green emission).

The sintered dispense-plotted assemblies had a characteristic mesh-like structure with rod diameters of 300±30 µm, and pore sizes between the rods of about 370±25 µm.. By measuring the geometrical density of the sintered scaffolds, a total porosity of about 60% was calculated. Relative density of the sintered samples determined by helium pycnometry was about 99% th.d., which indicates only a small amount of closed porosity inside the material rods. Two main material phases of the sintered ceramic were detected by semi-quantitative XRD measurements: 30% HA, 60% β-TCP. Also a slight peak of α-TCP (10%) was identified (Fig. S1). SEM micrographs of the specimens revealed the anisotropic inner structure of the ceramic scaffolds as a result of the layer by layer process of dispense-plotting (Fig. 3A). The scaffolds were easily loaded with oAEC as indicated by the low concentration of cells recorded in the cultural media at the end of the incubation periods, which never exceeded 0.01% of added cells (1×10^6^), as well as by the high concentration of oAEC recorded over the surface of the block bone substitutes (Fig. 3B).

### Sinus Augmentation Clinical Procedure

Animals did not show any post-operative complications nor clinical symptoms of maxillary sinusitis. Extra-oral surgical windows healed in all groups after 45 and 90 days p.i. Sinus explants appeared as uniform blocks of tissue with the scaffolds firmly inserted.

### Micro-CT Analysis

**Figure 4:**
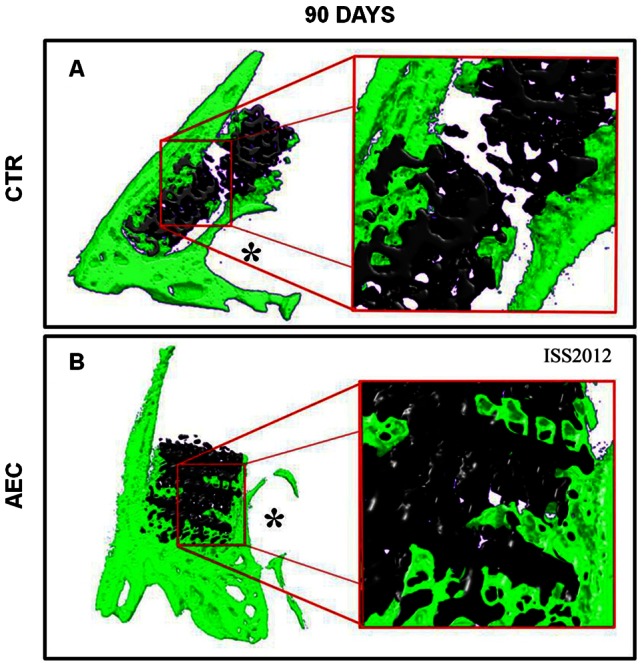
Micro-CT analysis: 3D reconstruction. Examples of 3D micro-CT analysis of sinus explants isolated after 90 days p.i. performed with the scaffold alone (CTR) or after engineering with oAEC (AEC). The different tissue densities were described by an arbitrary color scale showing in green and black colors the bone tissue and the biomaterial, respectively. *Asterisks indicate the infraorbital canal.

The 3D micro-CT analysis distinguished between bone tissue (green color) and bone substitute (black color) thus allowing to describe the process of bone generation and scaffold integration after sinus implants. The micro-CT revealed several newly deposited bone bridges, distributed between the native bone and the periphery of the scaffold in both CTR and oAEC. Foci of bone formation were also detected in the core of oAEC-treated sinuses differently from the CTR (Fig. 4).

### Histological Analysis

**Figure 5:**
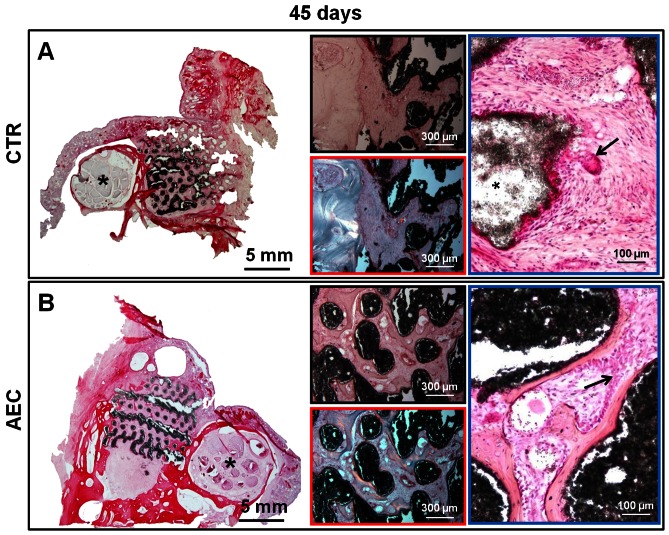
Morphological analysis of sinus explants 45 days p.i.. Examples of histological sinus explants cryosections stained with HE collected at 45 days p.i. performed with A) HA/β-TCP scaffold alone (CTR), and B) HA/β-TCP scaffold engineered with oAEC (AEC). Left big images: the lower magnification images show the whole implantation area demonstrating the integration of the scaffolds (dark structure) within the newly generated tissues. *Asterisks indicate the infraorbital canal. Middle images: in the top, representative examples of light images displaying the newly deposited matrix localized in the peripheral and central zones of CTR and oAEC-treated sinuses, respectively: in the bottom, polarized microscope images showing in blue and orange the immature and mature osteoid matrix, respectively. Right images: the higher magnifications show in CTR a multinucleate giant cell (top image: arrow) within the abundant fibrous connective tissue and in oAEC-treated explants, *trabeculae* of newly deposited bone tissue lined by a continuous layer of osteoblasts (bottom image: arrows).

**Figure 6:**
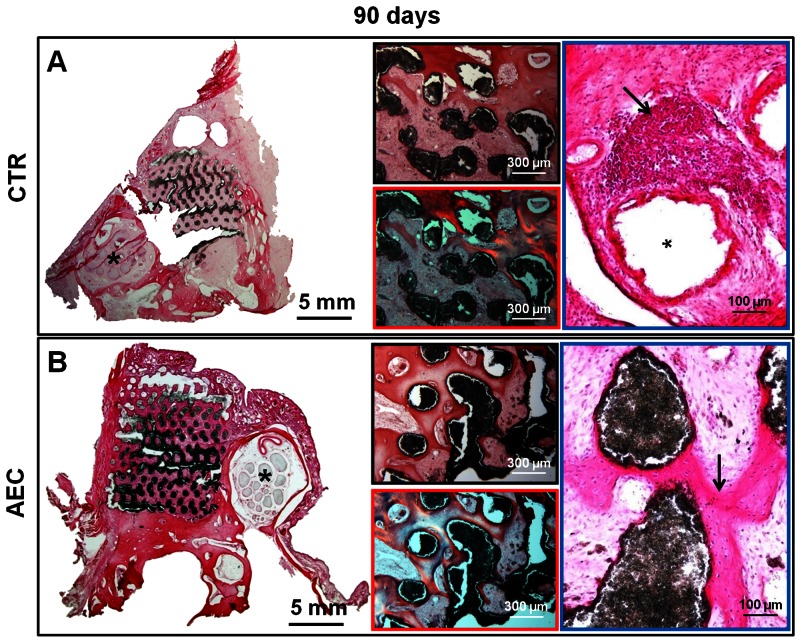
Morphological analysis of sinus explants 90 days after sinus lift. Examples of HE stained cryosections of sinus explants isolated at 90 days p.i. using A) HA/β-TCP scaffold alone (CTR), and B) HA/β-TCP scaffold engineered with oAEC (AEC). Left big images: the lower magnification shows the whole implantation area demonstrating the difference in bone matrix deposition. *Asterisks indicate the infraorbital canal. Middle images: in the top, light images displaying the foci of trabecular bone matrix localized in the peripheral zones of a CTR grafted sinus and the woven bone tissue distributed in the central area of an oAEC-treated sinus; at the bottom, polarized microscope images (bottom images) showing the higher level of maturation of newly deposited osteoid matrix recorded in oAEC-treated sinuses demonstrated by the prevalence of orange zones. Right images: the higher magnifications reveal in a CTR sinus (top image), beneath the *Schneiderian* membrane, a lymphoid follicle (top image: arrow) develops close to the scaffold cavity (top image: asterisk) surrounded by abundant fibrous tissue and newly deposited bone matrix. In oAEC-treated sinus, the presence of newly-formed bone tissue (bottom image: arrow) without evident inflammatory cell infiltrates was observed.

Scaffolds of CTR explants resulted constantly surrounded by fibrotic reaction. At 45 days p.i., a mild inflammation (score 1–2) was seen, mainly characterized by the presence of scattered “foreign-body type” multinucleate giant cells (Fig. 5A, black arrow). Then, the inflammation was from mild to moderate (score 1–2) and characterized by clusters of macrophages, usually containing scaffold particles, and to a lesser degree, by small aggregates of lymphocytes. The infiltration of lymphocytes, sometimes organized in follicle-like structures, was more prominent beneath the *Schneiderian* membrane, where the “mucosa-associated lymphoid tissue” is found constitutively (Fig. 6A, black arrow).

In oAEC-transplanted animals, fibrous tissue combined with newly deposited bone foci were found either at 45 or 90 days p.i.. Inflammation was absent or very mild (score 0–1) in both interval points and, almost exclusively supported by macrophages (Fig. 5 and 6).

As shown in Fig. 5 and Fig. 6, a good integration of scaffolds was always obtained. In oAEC-treated animals, foci of newly deposited bone were observed in the center and at the periphery of the scaffold (Fig. 5B and 6B). In CTR, the newly deposited bone became evident at the periphery of the grafted area only at 90 days p.i. (Fig. 6A). Osteogenesis was generated, in both groups, by the endosteal apposition of bone tissue deposited next to the native bone or the scaffold surface (Fig. 5 and 6). More in detail, *trabeculae* of bone tissue lined by a continuous layer of osteoblasts were clearly observed at 45 days p.i. in oAEC-treated animals (Fig. 5B). Bone foci were then diffusely observed in the core and at the periphery of implanted areas (Fig. 6B). Polarized light microscopy showed that the woven deposited bone (Fig. 6) recorded 90 days after oAEC transplantation is characterized by an abundant osteoid matrix (Fig. 6). By contrast, the foci of bone tissue, visualized mainly at the periphery of CTR sinuses at 90 days p.i. displayed an osteoid matrix with a different degree of maturation (Fig. 6 blue and orange areas). Noteworthy, the epithelial surface of the *Schneiderian* membranes was undamaged and formed by a continuous layer of ciliated cells, both in CTR and oAEC treated sinuses.

### Ovine AEC Detection In Sinus Explants

**Figure 7:**
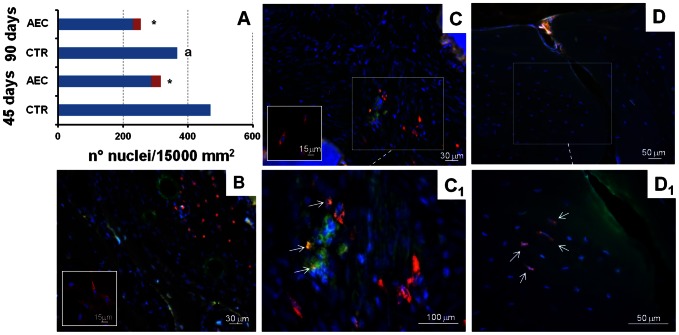
Detection of oAEC in transplanted sinuses. A) Histogram indicates the number of endogenous (blue bars) and transplanted cells (red bars) recorded in allotransplanted sinuses at 45 and 90 days p.i.. Endogenous cells or oAEC were identified by the DAPI counterstained nucleus visualized alone or surrounded by PKH26 dye, respectively. The values are expressed as mean. The SD never exceeded the 4%. ^*^ Values of total cells statistically different in oAEC *vs* CTR groups within the same p.i. interval, *p*<0.05 **^a^** Values of total cells recorded in oAEC or CTR groups significantly different when compared at 45 *vs* 90 days p.i., *p*<0.05; B) PKH26 labelled oAEC (red fluorescence) recorded in grafted sinuses at 45 days p.i.. The cells were recorded amongst fibrous matrix containing several endogenous cells identified by the bleu nuclei (DAPI fluorescence), newly deposited bone identified by the autofluorescence, blood vessels visualized by the endothelial marker, vWF (green fluorescence). Note how neither PKH26 labelled oAEC before (small box) and after transplantation express vWF. C-C_1_) PKH26-labelled oAEC (red fluorescence) and endogenous cells recorded in grafted sinuses at 90 days p.i.. The cells were recorded within the fibrous tissue at lower (C) and higher magnification (C_1_). Some of them are able to express OCN (green fluorescence), a typical bone-related protein. The OCN positivity was observed both in endogenous cells identified by the counterstained blue nuclei (DAPI) and in PKH26 positive cells (white arrows) that assume a typical orange fluorescent merge (green *plus* red). The PKH26 cells before transplantation did not express OCN (small box in C). D-D_1_) Osteocytes embedded within the newly deposited bone tissue observed at lower (D) and higher magnification (D_1_). The bone tissue, identified by the autofluorescence (green autofluorescence) of its matrix, showed several osteocytes. The blue nuclei (DAPI) identified the endogenous cells and the PKH26 dye displaying a typical spot fluorescence localized around the blue nuclei (arrows) identified oAEC transplanted cells.

**Figure 8:**
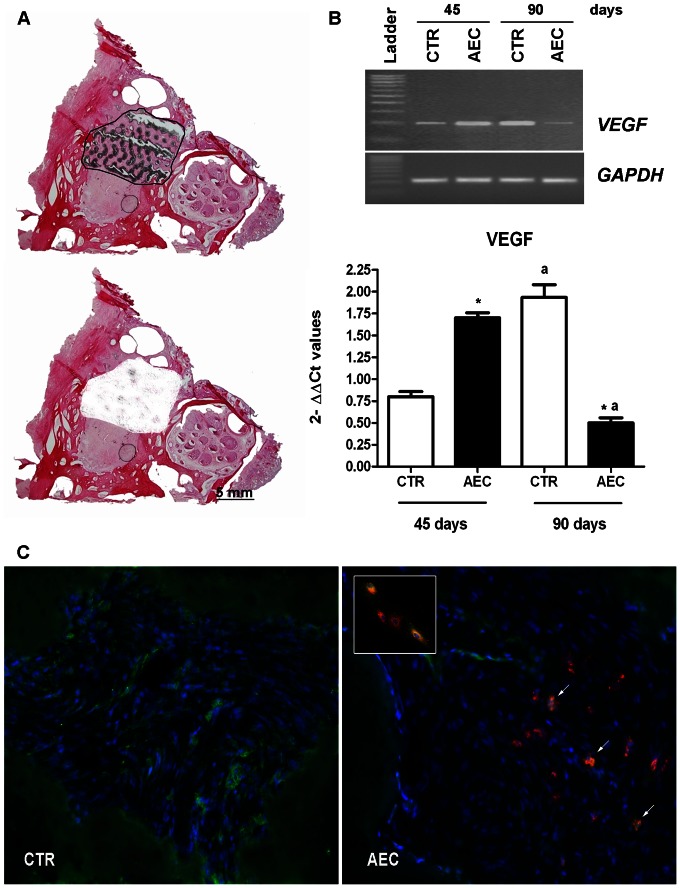
VEGF expression in grafted sinuses. A) Laser capture micro dissection (LCM) isolation of the implanted area from sinus explant. The top image shows an example of cryosection stained with HE where a black line delimits the implanted area that must be isolated by using LCM technique. The bottom image shows the same section after the implanted site was removed by LCM. Scale bar = 5 mm. B) VEGF mRNA expression. Top: image is a representative example of VEGF amplification. Bottom: quantitative VEGF analysis expression performed in real time PCR (values are mean of 3 replicates ±SD). *indicates values significantly different between CTR and oAEC groups within the same time interval (*p*<0.01), ^a^ indicates values significantly different in CTR or oAEC sinuses between the two time intervals (*p*<0.01). C) VEGF protein distribution in grafted sinuses 90 days after p.i.. Two representative images displaying the VEGF protein (green fluorescent signal) within the implanted area of a CTR (left image) and oAEC-treated sinuses (right image) explanted 90 days p.i. The nuclei are counterstained with DAPI in bleu and PKH26 labeled cells are visualized by the red fluorescence. Arrows indicate oAEC transplanted cells expressing VEGF (merge = orange fluorescence). Scale bars = 40 µm. The small box in the corner shows a representative example of VEGF expressing oAEC before grafting. The VEGF content of PKH26 labeled oAEC were analyzed on plated cultured cells. The merged orange fluorescence indicates the co-presence of PKH26 dye (red fluorescence) with the Alexa Fluor488 conjugated secondary antibody (green fluorescence). Scale bar = 60 µm.

Grafted oAEC were steadily recovered up to the 90^th^ day (*p*>0.05: Fig. 7A). In detail, oAEC represented, in both the experimental intervals, ∼10% of total cells (total cells = . endogenous cells plus PKH26 positive cells). On the contrary, the total cells recorded in CTR tissues at day 45 were significantly greater than those visualized at day 90 p.i. (*p*<0.05; Fig. 7A). Interestingly, oAEC transplantation was able to affect tissue cellularity: in fact, total cells detected in CTR groups were significantly greater than those recorded in oAEC-treated sinuses in both intervals (*p*<0.05 CTR *vs* oAEC at day 45 and *p*<0.05 CTR *vs* oAEC at day 90: Fig. 7A). Scattered isolated oAEC or, more frequently, oAEC aggregates were observed in proximity of blood vessels although none of them co-expressed vWF (Fig. 7B). By contrast, some PKH26 labeled cells displayed a cytoplasmic immune reactivity for OCN (Fig. 7C and C_1_), a specific bone related protein unexpressed in oAEC before transplantation (Fig. 7C, small box). OCN-immune reactivity was clearly detected in oAEC interspersed within the fibrous tissue (Fig. 7C_1_), but it was undetectable within the bone matrix because of the intense autofluorescence. PKH26-stained oAEC were also observed within the newly deposited bone matrix (Fig. 7D and D_1_) close to endogenous osteocytes. In order to evaluate the angiogenic role exerted by transplanted oAEC, VEGF distribution was also analyzed. Immunohistochemistry revealed that more than 50% oAEC displayed the ability to synthetize the angiogenic factor (Fig. 8C small box). VEGF/PKH26 co-localization was then, visualized in ∼35% and in ∼15 % of transplanted oAEC recorded at 45 (data not shown) and at 90 days p.i. (Fig. 8C arrows), respectively. Moreover, VEGF positivity displayed, first, a widespread distribution in oAEC-treated sinuses (data not shown) to become mainly surrounding the blood vessels at 90 days p.i. (Fig. 8C). Differently, VEGF positivity was very faint initially in CTR tissues (45 days, data not shown) while it increased at day 90 p.i. when it assumed a diffused distribution (Fig. 8C).

### Morphometric Analysis

**Figure 9:**
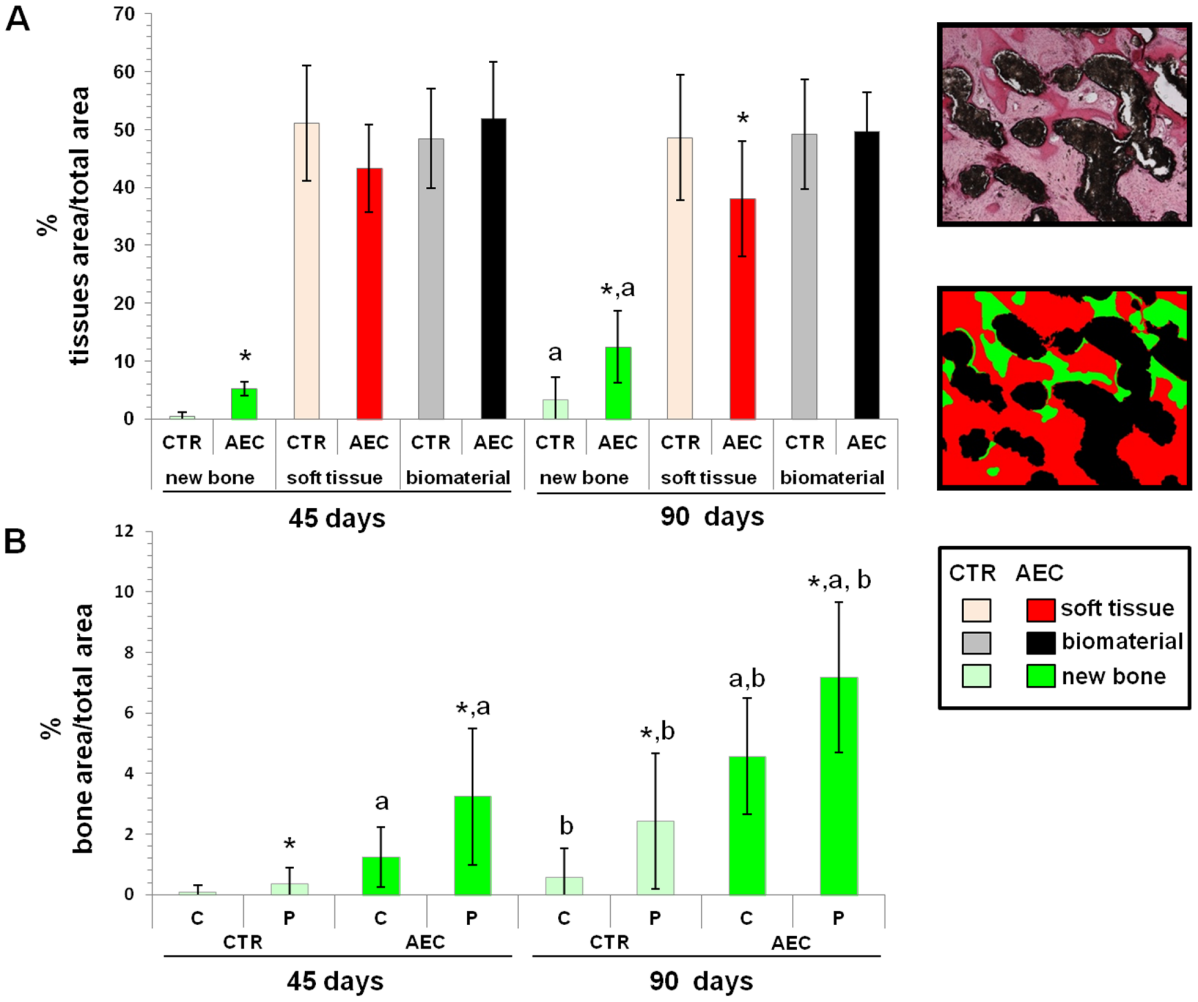
Morphometric analysis of grafted sinuses. A) Morphometric analysis of bone, biomaterials and fibrous tissues (soft tissue) area recorded within the grafted sinuses. The two images placed in the right corner show the digital conversion of the HE stained image used to quantify tissues/biomaterial area (green, new bone; red, fibrous tissue, and black, biomaterial) reported in the histograms. The data are expressed as mean ± SD. *Asterisks indicate values that resulted significantly different (*p*<0.05) when the two experimental groups were compared within the same interval point (i.e. CTR 45 days p.i. *vs* AEC 45 days p.i.). ^a^ letter indicates data significantly different (*p*<0.05) when area values at the different interval times within each experimental group were compared (i.e. AEC 45 days p.i. *vs* AEC 90 days p.i.). B) Histogram showed the morphometric data of newly deposited bone tissue recorded in the central (C) and peripheral zones (P) of grafted areas at 45 and 90 days p.i.. The data are expressed as mean ± SD. *Asterisks indicate C and P bone area values of CTR and AEC explants significantly different (*p*<0.05) when compared at each interval point (i.e. C AEC 45 day p.i. *vs* P AEC 45 days p.i.). ^a^ letter indicates C and P bone area values significantly different (*p*<0.05) when compared between the two experimental group at each interval point (i.e. P AEC 45 days p.i. *vs* P CTR 45 days p.i.). **^b^** letter indicate C or P bone area values recorded in CTR or oAEC-treated sinuses significantly different (*p*<0.05) when comparing the two interval points (i.e. C CTR 45 days p.i. *vs* C CTR 90 days p.i.).

The process of bone deposition progressively increased after sinus lift in both groups (Fig. 9A) even if the whole bone area always resulted significantly greater in oAEC-treated sinuses. The largest area of deposited bony tissue was recorded in oAEC grafted sheep at 90 days p.i., when a, parallel, reduction in fibrous tissue was observed (Fig. 9A). Newly formed bone was mainly localized in the periphery of the scaffold (Fig. 9B) both in oAEC and CTR sinuses. However, osteogenesis, resulted more diffuse in oAEC-treated sinuses where a greater extension of bony foci was observed in the central zones in both the interval times (Fig. 9B).

**Figure 10:**
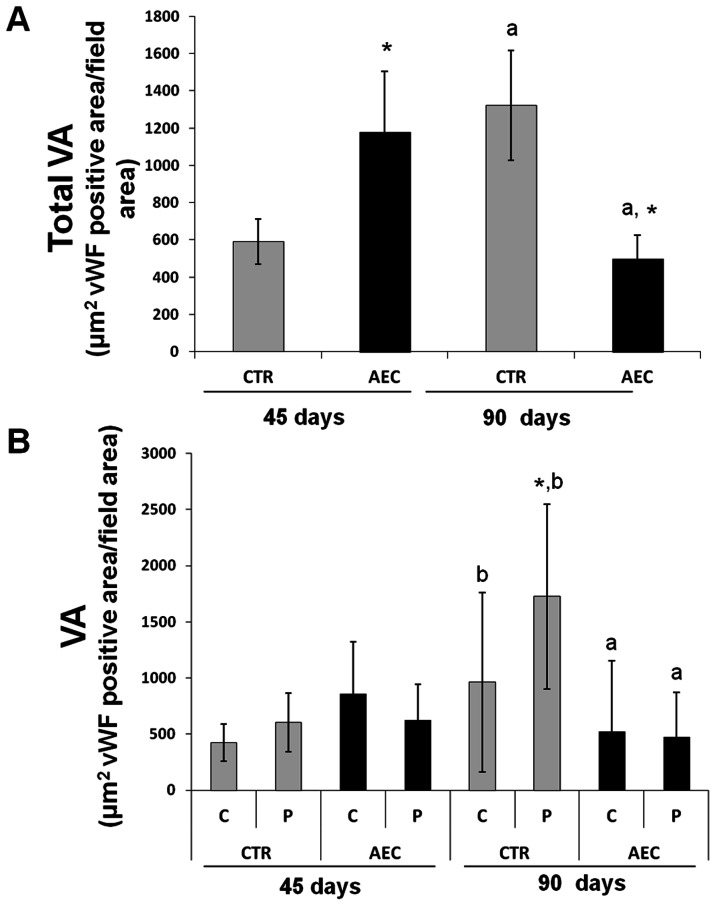
Morphometric analysis of vascular area (VA) in grafted sinuses. A) Morphometric analysis of total VA recorded in CTR and oAEC-grafted sinuses at 45 and 90 days p.i.. The histograms show the total VA quantified after the identification of blood vessel walls performed by using the vWF endothelial marker. The values of total VA were expressed as mean ± SD. *Asterisks indicate CTR *vs* AEC VA, significantly different (*p*<0.05) when recorded at each interval point (i.e. CTR 45 days p.i. *vs* AEC 45 days p.i.). ^a^ letter indicates values of VA recorded within each experimental group that resulted significantly different (*p*<0.05) when comparing the two time intervals (i.e. CTR 45 days p.i. *vs* CTR 90 days p.i.). B) Histogram showing VA recorded in the central (C) and peripheral (P) zones of grafted area analyzed at 45 and 90 days p.i.. The VA are expressed as mean ± SD. *Asterisks indicate C and P VA recorded in CTR or oAEC-treated sinuses significantly different (*p*<0.05) when compared at each interval point (i.e. C AEC 45 days p.i. *vs* P AEC 45 days p.i.). ^a^ letter indicates C or P VA recorded in CTR *vs* AEC significantly different (*p*<0.05) when compared at each interval point (i.e. P AEC 45 days p.i. *vs* P CTR 45 days p.i.). ^b^ letter indicates C or P VA recorded in CTR or oAEC-treated sinuses significantly different (*p*<0.05) when comparing the two interval points (i.e. C CTR 45 days p.i. *vs* C CTR 90 days p.i.).

Grafted oAEC significantly affected the extension of VA that resulted significantly greater at 45 days. (Fig. 10A) and lower at 90 days p.i.. By contrast, VA progressively increased in CTR group, thus gaining significantly higher values than those recorded in oAEC-treated animals at 90 days p.i. (*p*<0.01 Fig. 10A). Consistent with the pattern of bone deposition, topographic analysis of VA showed that angiogenesis was more uniform in oAEC–treated sinuses (Fig. 10B) than in CTR, where it was mainly localized in the periphery of the scaffold, in particular at 90 days p.i. (Fig. 10B).

The angiogenic response in the host tissue was studied, in addition, by analyzing the mRNA VEGF expression extracted from the implanted area selectively isolated with the aid of LMC techniques as summarized in Fig. 8A. The VEGF mRNA levels (see Fig 8) were significantly higher in oAEC than in CTR transplanted sinuses at day 45 p.i. (oAEC 45 *vs* CTR 45 days *p*<0.01) and became lower later (oAEC 90 *vs* CTR 90 days *p*>0.05). Consistent with VA results, in fact, the VEGF expression progressively increased in CTR grafted sinuses (CTR 45 *vs* 90 days, p<0.01) while it progressively decreased in the presence of oAEC (oAEC 45 *vs* 90 days *p*<0.01).

## Discussion

The clinical outcome of dental implants strongly depends upon the regeneration of bone tissue, which should be quali-quantitatively adequate and rapidly produced. To achieve those results, two technologies should be strictly integrated: biomaterial manufacturing and regenerative medicine. Previous and extensive studies demonstrated that chemical composition and physical structure of biomaterials are both crucial for cell ingrowth and bone deposition [72]–[74]. Our data confirm the high biocompatibility of HA/TCP ceramic scaffold made using rPT technique [15], [16]. In addition, the scaffold demonstrated to be an ideal substrate to support oAEC loading, thus facilitating the delivery of cells into the grafted sinuses. The scaffold used in our investigations reached a high degree of integration at 90 days p.i., but it was unable to stimulate a widespread process of bone deposition as demonstrated by the micro-CT and morphometric analyses.

Stated the poor osteo-regenerative properties induced by scaffolds transplantation, the *in vivo* osteogenic attitude of oAEC was easier to evaluate. On the basis of the present results, oAEC could be considered as a useful source of progenitor cells able to conjugate a good mesenchymal plasticity [25], [44], [46], [48], [49] with other useful biological properties. In fact, AEC are uncontroversial and largely available, are stable and can be easily expanded *in vitro*, thus overcoming the practical limitation still linked to the use of the mesenchymal stromal cells (MSC) isolated from adult tissues such as dental pulp, periodontal ligament, dental follicle and bone marrow aspirates [20], [22], [23], [75]–[82]. Furthermore, oAEC confirmed pro-angiogenetic activity and no tumurogenic attitude [29], [44], [47], [54], [83].

oAEC easily colonized the bone substitute when cultured under dynamic conditions and significantly improved bone tissue regeneration after *in vivo* allotransplantation. Both micro-CT and histological analysis demonstrated that the process of bone deposition was accelerated and enhanced by oAEC starting to identify the mechanisms involved.

oAEC were never shown to express endothelial markers vWF and, as a consequence, they apparently do not directly contribute to neo-angiogenesis. Notwithstanding this, oAEC showed a modulatory effect on neoangiogenesis, which could be mediated by their ability to secrete VEGF [26], [46] as well as by their modulatory effect on the VEGF expression of host maxillary tissues.

By contrast, molecular and morphological evidence suggests a direct role of oAEC in the process of osteogenesis. In fact, oAEC transplanted into the maxillary sinus acquired the ability to express OCN, a typical bone related protein, which is completely absent in cells before transplantation. A direct contribution of oAEC to the process of bone deposition may be, moreover, confirmed by the evidence that some PKH26 positive cells were detected within the newly deposited bone matrix. In fact, the typical red spots of PKH26 fluorescence surrounding flattened nuclei were recorded within bone matrix close to other endogenous osteocytes. However, beside a direct oAEC commitment into osteoblast lineage, the present results also suggest a paracrine role of oAEC involved in supporting tissue repair/regeneration. In fact, oAEC modulated the process of tissue regeneration by influencing either the cellularity or the inflammatory response in the transplanted sinuses. This last finding confirmed the immunomodulatory and a long term antifibrotic activity already demonstrated for the amniotic derived cells in another tissue model [84]. In particular, fibrous tissue in grafted oAEC sinuses was positively substituted after 90 days p.i by the newly deposited bone. Sinus explants revealed that the scaffolds themselves induced mild to moderate inflammatory reactions, characterized by the infiltration of clusters of macrophages and small lymphocytic aggregates always associated with a severe surrounding fibrotic reaction. In oAEC grafted sinuses, the fibrous tissue remained localized along the newly-formed *trabeculae* of bone tissue and the inflammation response was absent or very mild. The anti-inflammatory and anti-fibroblastic role exerted by the transplanted oAEC may have exerted a positive influence on the regenerative processes. Our scaffolds were fabricated to obtain a low degradation rate and robust mechanical property. However, as a consequence of its high porosity, the scaffold presents a large surface that, interacting with the host tissue, may potentially accelerate degradation due to macrophages via oxidation and/or hydrolysis. However, differences in scaffold degradation did not become evident in 90 days p.i. even if a higher infiltration of inflammatory cells/macrophages was observed in CTR tissues. The timing of scaffold degradation remains still to be defined since probably it requires longer periods than 90 days according to previously described tests [85], [86]. Ideally, scaffold degradation rate must be appropriately combined with the growth rate of the new-tissue, in such a way that the scaffold will be totally degraded by the time the implantation site is totally regenerated.

The oAEC influence on neoangiogenesis is more evident. By a simple histological examination, the scaffolds appeared completely colonized by a densely vascularized tissue in both experimental groups starting from 45 days p.i.. The morphometric analysis, however, revealed that the process of blood vessel rearrangement was significantly enhanced by oAEC at the beginning, to subsequently decline on values lower than in CTR. oAEC, in addition, stimulated a diffused vascular network that resulted equally distributed between the core and the periphery of the implanted area. This blood vessel organization may support a stable and homogenous trophic supply, thus increasing the more uniform bone deposition within the grafted sinus. By contrast, the VA progressively increased in the CTR explants, thus becoming significantly greater after 90 days p.i., mainly at the periphery of the grafted area. This different angiogenic behaviour may be interpreted as a consequence of the higher inflammatory reaction and VEGF expression recorded in CTR tissues at 90 days.

These results clearly indicate that the surgical outcome of a common approach such as the sinus augmentation procedure could be markedly improved by combining the innovative techniques in producing bone graft substitutes with the use of an adequate source of progenitor cells. Since bone regeneration proceeds always centripetal after the transplantation of a good synthetic bone substitute, the use of an adequate source of progenitor cells such as oAEC is determinant to enhance and make more extent the foci of bone nucleation, thus increasing, strengthening and accelerating the alveolar bone reconstruction.

Although the role of oAEC remains unclear and questionable, their transplantation clearly supported an overall increase of bone deposition either directly contributing to osteogenesis or indirectly modulating the major mechanisms (inflammation, cellularity and angiogenesis) involved in tissue regeneration. Both these mechanisms have also been previously described in bone marrow derived MSC as well as in other MSC sources [20]–[22], [75]–[77], [80]–[81] where, however, the reduced availability of progenitor cells still remains overcome problem. Actually, in the absence of experimental studies addressed to compare the regenerative properties of MSC *vs* AEC any speculation becomes difficult even if the results obtained in the present research together with the high biological properties of amniotic derived cells [28], [29] seems to suggest their potential relevance in dentistry.

In particular, the ability of oAEC in modulating the angiogenic remodeling and in reducing inflammatory reaction may be essential to drive crucial mechanisms involved in improving scaffold persistence and integration within the host tissue, as well as to guarantee an adequate trophic support to the different cells actively engaged in the process of tissue regeneration. Altogether, the present results provide the first evidence in favor of an efficient and safe therapeutic role of AEC. These results support the hypothesis that AEC hold much promise for the development of cell-based therapies in craniofacial surgical applications, leading to the idea of their safe use under allotransplantation settings.

## Supporting Information

Figure S1 **Biomaterial Characterization.** Semi-quantitative x-rays diffraction measurement of HA/β-TCP custom made scaffold showed that the bone substitute is composed of three co-existing mineral phases from the mineral phases point of view: β-TCP (60%), HA (30%) and αTCP (10%).
(TIF)
