# Supplementary figures and images for: Synthetic Bone Substitute Engineered with Amniotic Epithelial Cells Enhances Bone Regeneration after Maxillary Sinus Augmentation
Source: PLoS One. 2013 May 17;8(5):e63256. doi: 10.1371/journal.pone.0063256 (PMC3656960; doi:10.1371/journal.pone.0063256)

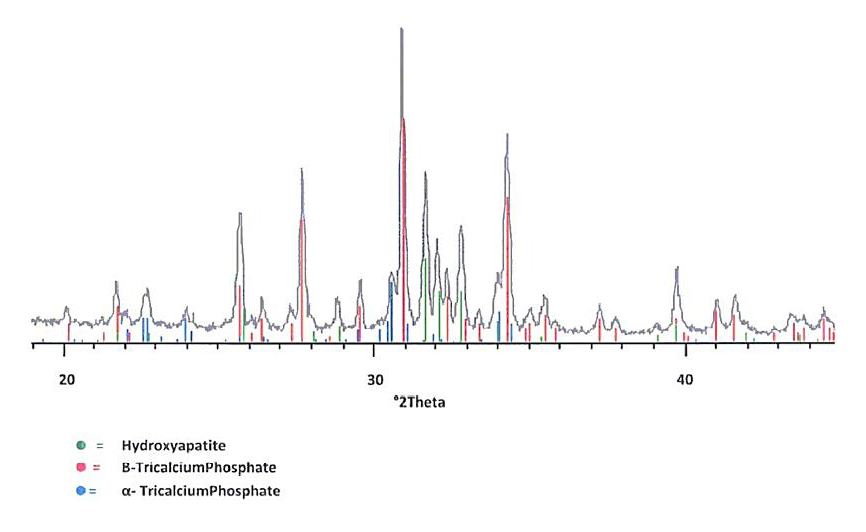

Supplement: Figure S1 — Biomaterial Characterization. Semi-quantitative x-rays diffraction measurement of HA/β-TCP custom made scaffold showed that the bone substitute is composed of three co-existing mineral phases from the mineral phases point of view: β-TCP (60%), HA (30%) and αTCP (10%). (TIF) [file pone.0063256.s001.tif]
